# Association of Poststroke Pain Prevalence and Subtypes With Lesion Sites in Ischemic and Hemorrhagic Stroke: A Cross-Sectional Study

**DOI:** 10.7759/cureus.115624

**Published:** 2026-09-01

**Authors:** Mizanur Rahman, Ahmed Hossain Chowdhury, Reaz Mahmud, S M Arafath Amin, Nishat Nayla Aurpa, Maksudur Rahman, Pijush Paul

**Affiliations:** 1 Department of Neurology, Dhaka Medical College Hospital, Dhaka, BGD; 2 Department of Neurology, Shaheed Syed Nazrul Islam Medical College, Kishoreganj, BGD; 3 Department of Neurology, National Institute of Neurosciences & Hospital, Dhaka, BGD; 4 Department of Interventional Neurology, National Institute of Neurosciences & Hospital, Dhaka, BGD

**Keywords:** central poststroke pain, hemiplegic shoulder pain, lesion site, poststroke pain, stroke

## Abstract

Background

Poststroke pain (PSP) is an underrecognized but clinically significant complication of stroke that adversely affects rehabilitation, mood, and quality of life. Evidence from Bangladesh and similar low- and middle-income settings examining PSP subtype frequency in relation to lesion characteristics remains limited. This study aimed to determine the prevalence and subtypes of PSP and to evaluate their associations with lesion site, lesion side, and stroke type among patients with ischemic and hemorrhagic stroke in Bangladesh.

Methods

This hospital-based cross-sectional study was conducted among 250 adult stroke patients who were recruited via purposive sampling and were attending the stroke clinic of Dhaka Medical College Hospital, Bangladesh, between two months and two years after the index stroke event, over a 24-month period between March 2021 and February 2023. Sociodemographic, clinical, and biochemical data were collected using a structured case record form. Pain intensity was assessed using the visual analog scale, and stroke type, lesion side, and lesion site were determined from computed tomography (CT) or magnetic resonance imaging (MRI) reports. Patients were evaluated for four PSP subtypes: central poststroke pain (CPSP), hemiplegic shoulder pain (HSP), contracture-associated pain (CAP), and poststroke headache. Multivariable binary logistic regression was used to assess factors independently associated with prevalent PSP; estimates are reported as adjusted prevalence odds ratios (AORs).

Results

PSP was identified in 104 (41.60%) patients. Among these 104 patients, poststroke headache was the most common subtype, 44 (42.30%), followed by CAP, 32 (30.80%); HSP, 25 (24.00%); and CPSP, 13 (12.50%). The corresponding prevalence rates in the total cohort were 17.60%, 12.80%, 10.00%, and 5.20%, respectively, and the subtypes were not mutually exclusive. CPSP and CAP were associated with right-sided lesions (p = 0.002 and p < 0.001, respectively), whereas headache was associated with the lesion site (p < 0.001). The factors independently associated with increased odds of prevalent PSP were low educational status (AOR = 2.305), right-sided lesions (AOR = 2.648), triglyceride level per 1 mg/dL (AOR = 1.015), and limited muscle power (AOR = 2.858); serum creatinine per 1 mg/dL was inversely associated (AOR = 0.263).

Conclusions

PSP was present in a substantial proportion of stroke survivors, with headache being the most common subtype. Lesion side, lesion site, educational status, muscle power, and biochemical markers were significantly associated with PSP, indicating a heterogeneous, subtype-specific phenomenon. Larger multicenter, longitudinal studies are needed to confirm these associations.

## Introduction

Worldwide, stroke continues to rank among the foremost contributors to mortality and long-term disability. The Global Burden of Disease 2021 study identified it as the third most common cause of death globally, accounting for 7.3 million deaths, 93.8 million prevalent cases, and 11.9 million incident cases in that single year [1]. In 2021, ischemic stroke alone was responsible for nearly 70 million prevalent cases and 70.4 million disability-adjusted life-years worldwide [2]. Beyond the risk of death, a large proportion of survivors are left with lasting neurological, functional, sensory, and pain-related consequences that hinder recovery and erode quality of life.

Poststroke pain (PSP) is a clinically meaningful yet frequently overlooked sequela that spans neuropathic, musculoskeletal, spasticity-related, and headache-related pain syndromes. Neuropathic pain of central origin constitutes only one element of this construct and has a pooled prevalence of approximately 11% [3]. How often the broader PSP construct is reported depends heavily on the diagnostic criteria applied, the timing of assessment, and which subtypes are counted [4,5]. Because PSP undermines rehabilitation, sleep, mood, mobility, and functional recovery as a whole, its early identification is central to comprehensive stroke care [5].

Among PSP subtypes, central poststroke pain (CPSP) arises from somatosensory tract injury and may be particularly common in thalamic or medullary strokes [3]. Hemiplegic shoulder pain (HSP), which is linked to subluxation, spasticity, and soft-tissue injury, affects approximately one-third of stroke survivors [6]. New-onset headache occurs in approximately 14% of ischemic stroke patients but in up to 46% of hemorrhagic stroke patients [7,8]. Spasticity-associated and contracture-related pain develops in 17%-43% of patients within one year poststroke [4].

Lesion characteristics have been associated with PSP occurrence, although the findings remain inconsistent. Thalamic involvement, particularly the ventroposterior nuclei, is strongly associated with CPSP [9], whereas spasticity-related pain has been linked to internal capsule, basal ganglia, and insular lesions [10]. Left-sided lesions are significantly associated with both CPSP and shoulder pain in pooled analyses [5]; however, evidence regarding lesion side and ischemic versus hemorrhagic stroke type as consistent correlates remains conflicting across studies.

The clinical factors associated with PSP subtypes include deep sensory dysfunction, younger age, greater stroke severity, and preexisting depression for CPSP [4,9] and reduced shoulder range of motion, spasticity, and impaired motor control for HSP [11]. Recognizing these features may support targeted clinical assessment, rehabilitation planning, pain management, and prevention of chronic disability. PSP research is especially pertinent in low- and middle-income countries (LMICs) such as Bangladesh, where the disease carries a considerable burden. A nationwide survey reported a stroke prevalence of 11.39 per 1,000 people, with ischemic stroke accounting for 79.7% and hemorrhagic stroke accounting for 15.7% of cases [7], whereas a hospital-based narrative review noted a comparatively greater proportion of hemorrhagic stroke than in Western cohorts [8]. Limited rehabilitation infrastructure, delayed follow-up, and underrecognition of pain symptoms further compound the burden in such settings [12].

Despite this, evidence from Bangladesh and similar LMIC settings specifically examining PSP subtype frequency in relation to lesion site, lesion side, and stroke type remains limited. This gap is clinically significant, as CPSP, HSP, contracture-associated pain (CAP), and poststroke headache (PSH) likely differ in mechanism, associated factors, and optimal management strategies, necessitating subtype-specific evaluation rather than aggregate PSP assessment. Generating locally relevant evidence could help clinicians recognize the true burden of PSP, identify lesion-related patterns specific to this population, and guide targeted follow-up and rehabilitation strategies for Bangladeshi stroke patients. Against this background, the present study set out to characterize the prevalence and subtypes of PSP and to examine how they relate to lesion site, lesion side, and stroke type in ischemic and hemorrhagic stroke patients in Bangladesh. The primary objective was to determine the prevalence and subtypes of PSP and their association with lesion site, lesion side, and stroke type; associations with sociodemographic, clinical, and biochemical factors were examined as exploratory, secondary objectives, consistent with the cross-sectional design and the backward stepwise variable-selection approach used in the multivariable analysis.

## Materials and methods

Study design and setting

This cross-sectional, hospital-based investigation was carried out in the Department of Neurology at Dhaka Medical College Hospital (DMCH), Dhaka, Bangladesh, spanning a 24-month interval from March 2021 to February 2023. DMCH is a tertiary-level teaching hospital that operates a dedicated stroke clinic offering comprehensive neurological care and neuroimaging services.

Study population

Participants were adults of either sex, at least 18 years of age, carrying a confirmed ischemic or hemorrhagic stroke diagnosis, who presented to the DMCH stroke clinic between two months and two years after their index stroke. This time frame was chosen to exclude the acute/subacute phase, during which pain of inflammatory, positional, or immobility-related origin is still evolving, and CPSP cannot yet be reliably diagnosed against established criteria, while remaining broadly consistent with intervals used in prior PSP studies to capture clinically established pain syndromes. Exclusion criteria comprised an index stroke of less than two months' duration, critical illness, global or receptive aphasia, pre-existing pain disorders, dementia, subarachnoid hemorrhage, transient ischemic attack, and unwillingness to give informed consent.

Sampling procedure and sample size

Participants were recruited through purposive sampling. Eligible patients attending the DMCH stroke clinic during the study period were identified by the investigators and approached as they presented. Those meeting the predefined inclusion and exclusion criteria were enrolled after providing written informed consent.

The sample size was calculated using Cochran's formula for estimating a single population proportion, as it is a standard method for determining the sample size in cross-sectional studies with a primary aim of estimating prevalence. The formula was as follows, where n is the required sample size, Zα is the standard normal deviate corresponding to the selected confidence level, P is the anticipated prevalence, and d is the absolute precision:

\[ n = \frac{Z_{\alpha}^{2}\,P(100-P)}{d^{2}} \]

Assuming a 95% confidence level (Zα = 1.96), an anticipated PSP prevalence of 21% derived from an earlier population-based study by Lundström et al. [13], and an absolute precision of 5% (d = 5%), the estimate yielded approximately 255 patients. Ultimately, 250 eligible participants were enrolled and analyzed during the study period, a modest shortfall of five from the intended target.

Data collection procedures

Information was captured using a structured case record form. Once written consent had been secured, comprehensive sociodemographic details, clinical history, and physical examination findings were documented. Pain intensity was rated on the visual analog scale (VAS), a validated 100-mm instrument commonly employed to quantify pain severity in clinical research [13], with scores classified as mild (VAS 5-44 mm), moderate (VAS 45-74 mm), or severe (VAS 75-100 mm). Muscle strength was graded with the Medical Research Council (MRC) scale. Stroke type (ischemic or hemorrhagic), lesion side (right or left), and lesion site (cortical, subcortical, or infratentorial) were ascertained from contrast computed tomography (CT) head or magnetic resonance imaging (MRI) brain reports available in the patient records. Pertinent laboratory parameters, namely the white blood cell (WBC) count, random blood sugar (RBS), serum creatinine, and lipid profile, were likewise recorded.

Assessment of PSP subtypes

Patients were systematically evaluated through structured interviews and clinical examinations for four major PSP subtypes: CPSP, HSP, CAP, and PSH. CPSP was diagnosed when pain occurred in a body area corresponding to a confirmed central nervous system lesion, began after the stroke, had features such as allodynia or hyperalgesia, and was not better explained by nociceptive or peripheral neuropathic pain, which is consistent with established CPSP criteria [14]. HSP was defined as new pain in the hemiparetic shoulder after stroke, accompanied by pain on movement and/or restricted range of motion and clinical evidence of subluxation or spasticity; pre-existing pain disorders were excluded through the eligibility criteria [15]. CAP was defined as pain localized to a joint or muscle group with clinically demonstrable fixed limitations of passive range of motion attributable to poststroke contracture. PSH was identified clinically from history and examination, including onset after stroke, characteristics, and relieving or precipitating factors; a formal International Classification of Headache Disorders, third edition (ICHD-3), distinguishing acute from persistent stroke-attributed headache was not applied. Patients can be classified into more than one PSP subtype because the subtypes are not mutually exclusive.

Statistical analysis

All records were checked for completeness and processed in the Statistical Package for the Social Sciences (SPSS) version 26 (IBM Corp., Armonk, NY). Continuous variables are summarized as means ± standard deviations and categorical variables as frequencies and percentages. Group comparisons relied on independent-samples t tests for continuous data and Pearson's chi-square test for categorical data whenever the expected-cell assumption held; the Fisher exact test was applied to sparse 2×2 tables and the Fisher-Freeman-Halton exact test to sparse 3×2 tables. Variables reaching statistical significance in the bivariable analyses, together with sex, educational status, diabetes mellitus status, hemiparesthesia status, MRC grade, lesion side, lesion site, and serum creatinine and triglyceride levels, were entered into a multivariable binary logistic regression model using backward stepwise selection, with the reported model comprising 250 participants. Given the high frequency of PSP (41.60%), the adjusted odds ratios are reported as adjusted prevalence odds ratios (AORs) rather than interpreted as prevalence ratios or risks, and all estimates are accompanied by 95% confidence intervals (CIs). Subtype analyses were regarded as exploratory, and a two-sided p-value < 0.05 defined statistical significance.

Ethical considerations

The study protocol received formal approval from the Ethical Review Committee (ERC) of Dhaka Medical College (approval no.: ERC-DMC/ECC/2021/49) and was carried out in keeping with the principles of the Declaration of Helsinki. Every participant provided written informed consent before enrollment. Patient data were handled with strict confidentiality and anonymity throughout, and participants were advised of their right to withdraw at any point without any repercussions.

## Results

Of the 250 stroke patients enrolled, 104 (41.60%) reported PSP, while 146 (58.40%) did not. Mean age was similar across the two groups (58.7 ± 11.8 years with pain vs. 59.5 ± 11.5 years without pain, p = 0.594), and the age distribution did not differ significantly (p = 0.266). Compared with the pain-free group, patients with pain were more often female (38 (36.50%) vs. 34 (23.30%), p = 0.023) and more often of low educational status (75 (72.10%) vs. 78 (53.40%), p = 0.003). Diabetes mellitus was more frequent among those without pain (85 (58.20%) vs. 43 (41.30%), p = 0.009), whereas hypertension, smoking, and cardiovascular disease showed no significant association. Hemiparesthesia occurred only in the pain group (13 (12.50%) vs. 0 (0.00%), p < 0.001), and limited motor function (MRC grade 0-3) was likewise more common among these patients (52 (50.00%) vs. 47 (32.20%), p = 0.005) (Table 1).

**Table 1:** Sociodemographic and clinical characteristics of stroke patients with and without poststroke pain (N = 250) The values are n (%) unless otherwise specified. Pearson's chi-square test was used for categorical variables with adequate expected counts, Fisher's exact test was used for sparse 2×2 comparisons, and an independent-samples t-test was used for mean age. *p < 0.05. SD: Standard deviation; MRC: Medical Research Council.

Variables	Without pain (N = 146)	With pain (N = 104)	p-value
Age (years)			
≤60 years	71 (48.6)	58 (55.8)	0.266
>60 years	75 (51.4)	46 (44.2)	
Mean ± SD	59.5 ± 11.5	58.7 ± 11.8	0.594
Gender			
Male	112 (76.7)	66 (63.5)	0.023*
Female	34 (23.3)	38 (36.5)	
Educational status			
Low (Illiterate–Primary)	78 (53.4)	75 (72.1)	0.003*
Secondary and above	68 (46.6)	29 (27.9)	
Comorbidities and risk factors			
Hypertension	124 (84.9)	92 (88.5)	0.422
Diabetes mellitus	85 (58.2)	43 (41.3)	0.009*
Smoking history	75 (51.4)	41 (39.4)	0.062
Cardiovascular disease	14 (9.6)	10 (9.6)	0.999
Clinical features			
Hemiparesis	142 (97.3)	101 (97.3)	0.999
Hemiparesthesia	0 (0.0)	13 (12.5)	<0.001*
Dysarthria	129 (88.4)	91 (87.5)	0.837
Motor function			
No or limited movement (0–3 MRC)	47 (32.2)	52 (50.0)	0.005*
MRC 4–5	99 (67.8)	52 (50.0)	

Ischemic stroke was the dominant type in both groups, with no significant difference by pain status (95 (91.30%) vs. 124 (84.90%), p = 0.129). Right-sided lesions were significantly more frequent in patients with pain (72 (69.20%) vs. 80 (54.80%), p = 0.021), and lesion site also differed significantly between the groups, infratentorial lesions being more common in the pain group (13 (12.50%) vs. 7 (4.80%), p = 0.047) (Table 2).

**Table 2:** Stroke characteristics of patients with and without poststroke pain (N = 250) *p < 0.05, chi-square test.

Stroke characteristics	Without pain (N = 146)	With pain (N = 104)	p-value
Type of stroke			
Ischemic	124 (84.9)	95 (91.3)	0.129
Hemorrhagic	22 (15.1)	9 (8.7)	
Side of lesion			
Right	80 (54.8)	72 (69.2)	0.021*
Left	66 (45.3)	32 (30.8)	
Site of lesion			
Cortical	73 (50.0)	41 (39.4)	0.047*
Subcortical	66 (45.2)	50 (48.1)	
Infratentorial	7 (4.8)	13 (12.5)	

On biochemical assessment, serum creatinine was significantly lower in patients with pain (1.1 ± 0.4 vs. 1.6 ± 1.4 mg/dL, p = 0.001). Triglyceride levels, in contrast, were significantly higher in this group (155.8 ± 58.5 vs. 140.4 ± 40.7 mg/dL, p = 0.023). The WBC count and the RBS, low-density lipoprotein (LDL), high-density lipoprotein (HDL), and total cholesterol values did not differ significantly between the groups (Table 3).

**Table 3:** Comparison of serum biomarkers between patients with and without poststroke pain (N = 250) The values are presented as means ± SDs. *p < 0.05, independent t-test. WBC: White blood cell; RBS: Random blood sugar; LDL: Low-density lipoprotein; HDL: High-density lipoprotein.

Biomarker	Without pain (N = 146)	With pain (N = 104)	p-value
WBC (×10³/mm³)	10079.4 ± 3256.1	10420.9 ± 3564.1	0.440
Creatinine (mg/dL)	1.6 ± 1.4	1.1 ± 0.4	0.001*
RBS (mmol/L)	9.5 ± 4.8	9.1 ± 5.6	0.526
LDL (mg/dL)	118.8 ± 19.9	122.5 ± 35.2	0.339
HDL (mg/dL)	43.5 ± 7.6	45.1 ± 9.7	0.181
Triglyceride (mg/dL)	140.4 ± 40.7	155.8 ± 58.5	0.023*
Total cholesterol (mg/dL)	187.9 ± 26.2	187.8 ± 42.7	0.992

Among the 104 patients with PSP, PSH was the most frequent subtype (44 (42.30%)), followed by CAP (32 (30.80%)), HSP (25 (24.00%)), and CPSP (13 (12.50%)). Subtypes were not mutually exclusive, as some patients experienced more than one (Table 4). Moderate pain was the predominant severity category for HSP (15 (60.00%)), CAP (17 (53.10%)), and PSH (18 (40.90%)), whereas CPSP was predominantly mild (7 (53.80%)) (Table 4).

**Table 4:** Frequency distributions of poststroke pain subtypes and predominant pain severity (N = 250) The percentage of patients with PSP was calculated using 104 as the denominator, whereas the percentage for the total cohort was calculated using 250 as the denominator. PSP: Poststroke pain.

PSP subtype	n (%) of 104 PSP patients	Predominant severity within subtype, n (%)
Central poststroke pain (CPSP)	13 (12.5)	Mild, 7 (53.8)
Hemiplegic shoulder pain (HSP)	25 (24.0)	Moderate, 15 (60.0)
Contracture-associated pain (CAP)	32 (30.8)	Moderate, 17 (53.1)
Poststroke headache (PSH)	44 (42.3)	Moderate, 18 (40.9)

CPSP was significantly associated with right-sided lesions (13 (100.00%) vs. 139 (58.60%) in those without CPSP, p = 0.003). CAP was significantly associated with cortical lesions (24 (75.00%) vs. 90 (41.30%), p = 0.001), ischemic stroke (32 (100.00%) vs. 187 (85.80%), p = 0.019), and right-sided lesions (29 (90.60%) vs. 123 (56.40%), p < 0.001). PSH was significantly more common with noncortical (subcortical and infratentorial) lesions and was notably less common with cortical involvement (5 (11.40%) vs. 109 (52.90%), p < 0.001). HSP was not significantly associated with stroke site, type, or side (Table 5).

**Table 5:** Associations of poststroke pain subtypes with the site, type, and side of the stroke lesion (N = 250) *p < 0.05. Pearson's chi-square test was used when the expected-cell assumption was met, Fisher's exact test was used for sparse 2×2 tables, and the Fisher–Freeman–Halton exact test was used for sparse 3×2 tables.

(A) Central poststroke pain (CPSP)			
Variable	Without CPSP (N = 237)	CPSP (N = 13)	p-value
Site of Lesion			
Cortical	110 (46.4)	4 (30.8)	0.509
Subcortical	108 (45.6)	8 (61.5)	
Infratentorial	19 (8.0)	1 (7.7)	
Type of Stroke			
Ischemic	206 (86.9)	13 (100.0)	0.379
Hemorrhagic	31 (13.1)	0 (0.0)	
Side of Lesion			
Right	139 (58.6)	13 (100.0)	0.002*
Left	98 (41.4)	0 (0.0)	
(B) Hemiplegic shoulder pain (HSP)			
Variable	Without HSP (N = 225)	HSP (N = 25)	p-value
Site of Lesion			
Cortical	104 (46.2)	10 (40.0)	0.618
Subcortical	104 (46.2)	12 (48.0)	
Infratentorial	17 (7.6)	3 (12.0)	
Type of Stroke			
Ischemic	195 (86.7)	24 (96.0)	0.332
Hemorrhagic	30 (13.3)	1 (4.0)	
Side of Lesion			
Right	140 (62.2)	12 (48.0)	0.167
Left	85 (37.8)	13 (52.0)	
(C) Contracture-associated pain (CAP)			
Variable	Without CAP (N = 218)	CAP (N = 32)	p-value
Site of Lesion			
Cortical	90 (41.3)	24 (75.0)	0.001*
Subcortical	110 (50.5)	6 (18.8)	
Infratentorial	18 (8.3)	2 (6.3)	
Type of Stroke			
Ischemic	187 (85.8)	32 (100.0)	0.019*
Hemorrhagic	31 (14.2)	0 (0.0)	
Side of Lesion			
Right	123 (56.4)	29 (90.6)	<0.001*
Left	95 (43.6)	3 (9.4)	
(D) Poststroke headache (PSH)			
Variable	Without PSH (N = 206)	PSH (N = 44)	p-value
Site of Lesion			
Cortical	109 (52.9)	5 (11.4)	<0.001*
Subcortical	87 (42.2)	29 (65.9)	
Infratentorial	10 (4.9)	10 (22.7)	
Type of Stroke			
Ischemic	184 (89.3)	35 (79.5)	0.074
Hemorrhagic	22 (10.7)	9 (20.5)	
Side of Lesion			
Right	128 (62.1)	24 (54.5)	0.349
Left	78 (37.9)	20 (45.5)	

In the exploratory multivariable model, low educational status (AOR = 2.305, 95% CI: 1.195-4.446, p = 0.013), right-sided lesions (AOR = 2.648, 95% CI: 1.368-5.125, p = 0.004), higher triglyceride levels per 1 mg/dL (AOR = 1.015, 95% CI: 1.007-1.022, p < 0.001), and limited muscle power (MRC 0-3) (AOR = 2.858, 95% CI: 1.530-5.340, p = 0.001) each showed an independent association with greater odds of prevalent PSP. Conversely, serum creatinine per 1 mg/dL was inversely related to the odds of prevalent PSP (AOR = 0.263, 95% CI: 0.115-0.600, p = 0.002) (Table 6).

**Table 6:** Factors independently associated with prevalent poststroke pain according to a multivariable logistic regression model (N = 250) Associations were estimated using a multivariable binary logistic regression model, and a two-sided p-value < 0.05 was considered statistically significant. AORs describe the odds of prevalent PSP and should not be interpreted as prevalence ratios or risks. The reference categories used for the outcome were as follows: secondary education or above, left-sided lesion, MRC score 4–5, and no PSP. Creatinine and triglyceride levels were modeled as continuous variables per 1 mg/dL. Model: Nagelkerke R² = 0.350. The model was selected via backward stepwise selection and should be considered exploratory. β: Regression coefficient; SE: Standard error; AOR: Adjusted prevalence odds ratio; CI: Confidence interval; MRC: Medical Research Council.

Variable	β	SE	Wald	AOR	95% CI	p-value
Low educational status	0.835	0.335	6.209	2.305	1.195–4.446	0.013
Right-sided lesion	0.974	0.337	8.348	2.648	1.368–5.125	0.004
Serum creatinine (per 1 mg/dL)	−1.336	0.421	10.053	0.263	0.115–0.600	0.002
Triglyceride (per 1 mg/dL)	0.015	0.004	15.998	1.015	1.007–1.022	<0.001
Low muscle power (MRC 0–3)	1.050	0.319	10.841	2.858	1.530–5.340	0.001

## Discussion

The present study detected PSP in a considerable proportion of stroke survivors, with PSH being the most frequent subtype, followed by CAP and HSP, and CPSP being the least frequent. Lesion side and lesion site, along with selected clinical and biochemical variables, were significantly associated with PSP, whereas stroke type (ischemic versus hemorrhagic) was not associated with pain status. Taken together, these results indicate that PSP in this Bangladeshi cohort behaves as a heterogeneous, subtype-specific entity rather than a uniform outcome of cerebrovascular injury, and that its associated factors extend beyond stroke type alone.

A notable finding was that 104 of the 250 patients (41.60%) exhibited some form of PSP, a proportion that falls within the wide range documented across various poststroke stages and settings. A multicenter hospital-based study reported an overall pain prevalence of approximately 30%, with marked variation across acute, subacute, and chronic stages [16]. The variation in reported PSP frequency likely reflects differences in definition, time from stroke to assessment, study setting, and pain subtypes evaluated [4,16]. The relatively high frequency observed here may partly reflect the inclusion of multiple overlapping subtypes rather than a single pain construct. This burden has important rehabilitation implications, as PSP has been shown to impair sleep, mood, participation, and overall quality of life, thereby potentially slowing functional recovery [17].

Another important observation was the differential distribution and severity of PSP subtypes. PSH was the most prevalent headache subtype in our cohort, which is broadly consistent with reviews reporting headache prevalence rates ranging from 6% to 44% among ischemic stroke survivors [17,18]. CAP and HSP, both of which are linked mechanistically to spasticity, immobility, and soft-tissue changes, are also common, in line with the literature describing HSP in up to 40% of stroke survivors and emphasizing its multifactorial neurological and mechanical origins [19,20]. The predominance of moderate-severe pain in patients with HSP, CAP, and headache suggests a clinically meaningful burden even though pain is not predominantly severe. CPSP is relatively uncommon, which is consistent with pooled prevalence estimates of approximately 11% in unselected stroke populations [3]. This lower frequency may reflect the strict clinical criteria required for CPSP diagnosis, exclusion of pre-existing pain conditions, and potential under-recognition of neuropathic symptoms in routine practice.

Lesion laterality and topography showed different associations across PSP subtypes. The exclusive occurrence of CPSP among patients with right-sided lesions differed from pooled evidence suggesting an association with left-sided lesions [5]. Given the small number of CPSP cases (n = 13) and the exclusion of patients with global or receptive aphasia, which may have disproportionately excluded left-hemispheric strokes, this laterality finding should be interpreted cautiously as potentially affected by selection bias. CAP was associated with cortical, ischemic, and right-sided lesions, but the small and zero cells and the absence of multiplicity adjustment make these findings exploratory. PSH was more common with noncortical lesions in this cohort. This differs from reports that cortical infarcts are more often accompanied by headache than subcortical or lacunar lesions [21,22], although posterior circulation and midbrain involvement have also been implicated [23]. The relationship between lesion topography and PSH therefore remains inconsistent and sensitive to case definition and assessment timing. HSP showed no significant lesion-specific association, which is consistent with its multifactorial relationship with motor impairment and soft-tissue mechanics rather than a single lesion location [15,20].

The exploratory multivariable model identified low educational status, right-sided lesions, higher triglyceride levels, and limited muscle power (MRC 0-3) as factors independently associated with higher odds of prevalent PSP, whereas serum creatinine showed an inverse association. These cross-sectional associations should not be interpreted as temporal predictors or used for risk stratification. Low education may reflect delayed care seeking, reduced health literacy, or limited access to rehabilitation rather than a direct biological pathway. Limited muscle power may be related to immobility, shoulder subluxation, spasticity, and altered sensorimotor processing, which contribute to HSP and CAP [24,25]. Triglycerides were modeled as a continuous variable per 1 mg/dL; therefore, the result does not establish an “elevated” clinical category. The triglyceride and creatinine findings may reflect residual confounding by vascular risk, body composition, muscle mass, nutritional status, or renal function and require confirmation in longitudinal studies.

This study is among the first from Bangladesh to evaluate multiple PSP subtypes in relation to lesion characteristics via structured clinical assessment. However, several limitations warrant consideration. The single-center, purposive sample limits generalizability, and enrollment fell slightly below the calculated target (250 vs 255). Because participants were identified and enrolled by investigators rather than through random or fully consecutive sampling, purposive sampling carries an inherent risk of selection bias, potentially favoring patients with more overt symptoms; excluding patients with global or receptive aphasia may additionally have introduced laterality-related selection bias. The cross-sectional design precludes causal or temporal inference. Although the two-month-to-two-year enrolment interval was chosen to exclude the acute/subacute phase, it still spans a period over which pain subtypes evolve. Exact time since stroke was not retained in a form permitting continuous adjustment or stratified analysis; therefore, this potential confounding could not be evaluated and should be captured explicitly in future studies.

Lesions were classified from routine imaging reports. Headache was not classified using ICHD-3 criteria, and CAP/HSP were assessed without goniometry, dedicated imaging, or standardized methods to distinguish competing musculoskeletal causes, risking misclassification that could bias associations toward or away from the null. PSP subtypes overlapped, but the number of patients with multiple subtypes was unavailable. Regression relied on data-driven variable selection without prespecified confounder adjustment or model diagnostics; given the common outcome, odds ratios may overstate the association magnitude, and possible skewness or outliers in creatinine could not be reassessed without raw data. Sparse cells and the absence of multiplicity adjustment mean that subtype and regression findings, including laboratory associations, should be considered exploratory and may reflect chance. Pain was self-reported via the VAS, which is subjective and prone to recall bias, and no data were collected on treatments that may have modified pain perception. Recruitment at a single tertiary center may have favored more severe cases, limiting generalizability to community settings.

## Conclusions

PSP was identified in a substantial proportion of stroke survivors in this study, with PSH emerging as the most frequent subtype, followed by CAP, HSP, and CPSP. Lesion side, lesion site, low educational status, limited muscle power, and selected biochemical parameters were significantly associated with pain occurrence, whereas overall stroke type was not significantly associated. These findings suggest that PSP is a heterogeneous, subtype-specific phenomenon rather than a uniform consequence of cerebrovascular injury. Larger multicenter, longitudinal studies incorporating standardized subtype definitions, time since stroke, and appropriate prevalence ratio models are needed to confirm these associations and clarify their implications for clinical assessment and rehabilitation planning.
